# Mediating Effects of Systemic Inflammation on the Association Between Body Roundness Index and Periodontitis in US Adults

**DOI:** 10.1016/j.identj.2025.04.012

**Published:** 2025-06-10

**Authors:** Hui Zhang, Zhengyun Ren, Xi Peng, Tailin Guo

**Affiliations:** a College of Medicine, Southwest Jiaotong University, Chengdu, Sichuan, China; b School of Materials Science and Engineering, Key Laboratory of Advanced Technologies of Materials Ministry of Education, Southwest Jiaotong University, Chengdu, Sichuan, China; c College of Life Science, Southwest Jiaotong University, Chengdu, Sichuan, China

**Keywords:** BRI, Periodontitis, Systemic inflammation, NHANES

## Abstract

**Introduction and Aim:**

Periodontitis is a highly prevalent chronic inflammatory disease affecting periodontal tissues. While the Body Roundness Index (BRI), has emerged as a novel anthropometric measure for evaluating obesity-related health risks, its relationship with periodontal health remains unexplored. Although systemic inflammation is recognized as a key role in both obesity and periodontitis. However, whether BRI affects periodontitis, the mediating role of systemic inflammation in BRI-related periodontitis has not been elucidated.

**Materials and Methods:**

Data were derived from the National Health and Nutrition Examination Survey (NHANES) 2009-2014, comprising 8415 participants aged ≥18 years. We analysed BRI's correlation with periodontal disease using binary logistic regression models. Restricted Cubic Spline (RCS) modelling explored nonlinear patterns. The predictive performance of BRI for periodontitis was compared with traditional anthropometric indices using Receiver Operating Characteristic (ROC) curves. Mediation models assessed how systemic inflammation (SII, SIRI) bridges the BRI-periodontitis link.

**Results:**

In the fully adjusted model, participants in higher BRI quartiles showed progressively increased odds of periodontitis compared with those in the lowest quartile (Q1), with odds ratios of 1.33 (95% CI: 1.07-1.65, *P* = .010) for Q2, 1.48 (95% CI: 1.16-1.88, *P* = .004) for Q3, and 1.70 (95% CI: 1.20-2.40, *P* = .010) for Q4. RCS analysis indicated a linear relationship between BRI and periodontitis risk (nonlinearity *P* = .201). ROC curves revealed that BRI demonstrated superior predictive performance for periodontitis compared to BMI. The mediation analysis indicated that SII (5.37%, 95% CI: 0.86%-15.02%, *P* < .001) and SIRI (8.92%, 95% CI: 2.73%-22.13%, *P* < .05) partially mediated the BRI-periodontitis association.

**Conclusions:**

Our study demonstrates that elevated BRI is positive associated with an increased risk of periodontitis. Systemic inflammation, as reflected by SII and SIRI, partially mediates this relationship.

**ClinicalRelevance:**

Body Roundness Index (BRI), has emerged as a novel anthropometric indicator that more precisely estimates visceral adiposity and body fat percentage. Obesity, particularly the dysfunction of adipose tissue in visceral obesity, leads to the secretion of many pro-inflammatory factors, triggering systemic inflammatory responses. Systemic inflammation is recognized as a key role in both obesity and periodontitis. However, whether BRI affects periodontitis, the mediating role of systemic inflammation in BRI-related periodontitis has not been elucidated. This study supports that BRI is significantly associated with an increased risk of periodontitis, and systemic inflammation partially mediates this relationship. These findings highlight the importance of addressing obesity and systemic inflammation as part of periodontal disease prevention and management strategies.

## Introduction

Periodontitis involves chronic inflammatory damage to periodontal tissues, progressively compromising dental structural integrity, potentially culminating in tooth loss.^1^ The prevalence of periodontitis among adults with natural dentition reached approximately 60% between 2011 and 2020. Globally, severe periodontitis affects over 1 billion people, imposing substantial psychological, social, and economic burdens.^2^^,^^3^ Accumulating evidence has established periodontitis as a pivotal risk factor substantial implications for multiple systemic conditions, including cardiovascular disease, diabetes, and adverse pregnancy outcomes, highlighting its broader implications for public health.^4^, ^5^, ^6^, ^7^ Effective prevention, early diagnosis, and multidisciplinary management are critical to mitigating the widespread effects of this chronic condition.

Body Mass Index (BMI), while widely used indicator for obesity assessment, has recognized limitations in characterizing body fat distribution and distinguishing between visceral and subcutaneous fat, primarily because it fails to differentiate between fat-free mass and fat mass.^8^^,^^9^ Similarly, waist circumference (WC) can assess abdominal obesity, it does not account for height differences and cannot accurately assess the impact of visceral fat.^10^ The Body Roundness Index (BRI), incorporating both waist circumference and height measurements, has emerged as a novel anthropometric indicator that more precisely estimates visceral adiposity and body fat percentage (BF%).^9^^,^^11^ This is particularly relevant, one significant hallmark of obesity is the dysfunction of adipose tissue, particularly in visceral obesity, where adipocytes secrete various pro-inflammatory cytokines, including Il-6, IL-1β, and TNF-α.^12^ These pro-inflammatory factors induce a persistent systemic inflammatory response through systemic circulation. The systemic inflammatory state involves pro-inflammatory factors in the blood acting on periodontal tissues via blood circulation, affecting their immune microenvironment and leading to an increase in inflammatory factors in the gingival crevicular fluid. This immune dysregulation exacerbates local inflammatory responses and damages periodontal tissues, thereby increasing the risk of periodontitis.^13^ Even in individuals with normal BMI, the presence of excessive visceral fat may still increase periodontal disease risk.^14^ Based on this, BRI, as an accurate indicator of visceral fat, may indirectly predict the development of periodontitis through this inflammatory mechanism. While the obesity-periodontitis relationship is well-documented, conventional anthropometric measures inadequately capture the specific impact of fat distribution patterns on periodontal health.^15^ BRI has demonstrated promising predictive value for various metabolic outcomes, including diabetes and cardiovascular disease.^16^^,^^17^ However, its relationship with periodontal disease remains unexplored.

Systemic Inflammation, characterized by persistent inflammatory responses throughout the body, plays a pivotal role in various pathological conditions. The systemic immune-inflammation index (SII) and systemic inflammatory response index (SIRI) has emerged as novel, integrated biomarkers that comprehensively assess systemic inflammatory status by incorporating multiple cellular components, including platelet, neutrophil, monocyte, and lymphocyte.^18^ These indices, readily calculated from routine haematological parameters, have demonstrated significant associations with both periodontitis, and BMI (obesity) has been shown to affect SII and SIRI.^19^^,^^20^ Thus, systemic inflammatory status may mediate the relationship between body indices and periodontitis.

Despite growing evidence linking obesity with periodontal inflammation, the specific role of BRI—a more precise measure of body fat distribution—and its potential inflammatory mechanisms remain unexplored. Therefore, using data from the National Health and Nutrition Examination Survey (NHANES), we aimed to investigate the association between BRI and periodontitis, with particular focus on the mediating effects of systemic inflammatory markers (SII and SIRI) in this relationship.

## Methods

### Study design and participants

NHANES is a national cross-sectional study organized by the National Center for Health Statistics (NCHS) to assess the health and nutritional status of the noninstitutionalized population in the United States. The survey uses a precise stratified, multistage probability sampling design to ensure the representativeness and reliability of the samples. All participants are required to provide informed consent. All data are publicly available through NCHS and can be accessed via the official website (https://www.cdc.gov/nchs/nhanes/).

**Fig. 1:**
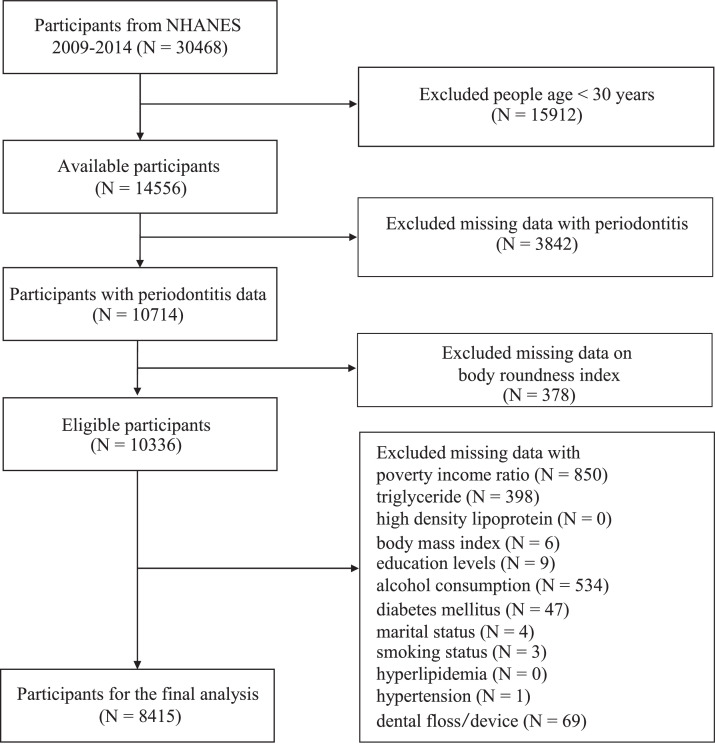
Flowchart of procedures for participants selection and inclusion.

This analysis utilized NHANES data from 2009-2014, the period when Full Mouth Periodontal Examination (FMPE) was exclusively conducted. From an initial sample of 30,468 participants, we applied several exclusion criteria: participants aged below 30 years (N = 15,912) were excluded as FMPE was restricted to adults ≥30 years; those with missing data on periodontitis (N = 3842) or BRI (N = 378); and participants lacking information on essential covariates (N = 1921). The final analytical sample comprised 8415 participants, representing a weighted population of 118,706,003 individuals (Figure 1).

### Definition of BRI and ABSI

BRI, a novel anthropometric measure, integrates height and waist circumference to evaluate body shape and composition. Anthropometric measurements were conducted by professional technicians at mobile examination centres (MEC) following standardized protocols. Height and waist measurements were obtained under standardized anthropometric conditions. BRI was then computed via a predefined formula^2^:

$$BRI=364.2-365.5\times \sqrt{1-{\left(\frac{WC}{2\pi }\right)}^{2}/{\left(0.5\times height\right)}^{2}}$$

ABSI (a body shape index): The ABSI is an indicator of obesity that contains multi-dimensional measurements including waist circumference data to assess body fat distribution. ABSI is calculated using the following equation^21^:

$$\text{ABSI}=\frac{WC\left(m\right)}{BMI{\left(\frac{kg}{m^{2}}\right)}^{\frac{2}{3}}\times height{\left(m\right)}^{\frac{1}{2}}}$$

### Assessment and classification of periodontal status

Periodontal examinations were conducted during the NHANES 2009-2014 cycles on participants aged ≥ 30 years who met the following criteria: presence of at least one natural tooth and no recent antibiotic use. The examinations were performed by calibrated dental hygienists following standardized protocols established by the Centers for CDC and the AAP. The periodontal assessment included measurements of probing depth (PD) and clinical attachment level (CAL) at 6 sites per tooth. Based on these clinical parameters, periodontal disease was classified according to the CDC/AAP case definitions (Table S1).^3^ For analytical purposes, participants were stratified into binary diagnostic groups: 1 representing periodontal health (“no periodontitis”) and 1 representing a comprehensive periodontitis group (mild, moderate, or severe; “periodontitis”).

### Definition of SII and SIRI

Although C-reactive protein is a commonly used systemic inflammation biomarker, due to the absence of C-reactive protein data for the 2011-2014 NHANES cycles, we chose to use SII and SIRI, which are also reliable indicators of systemic inflammation. Complete blood count (CBC) analyses were performed on participants' blood samples at NHANES mobile examination centres using the Beckman Coulter DxH 800 automated haematology analyser. The analyser quantified cellular components including lymphocytes, neutrophils, monocytes, and platelets, with results reported in 10^3^ cells/mL. The study assesses systemic inflammation by calculating the SII and SIRI using the following formulas: SII = (platelet count × neutrophil count)/lymphocyte count, and SIRI = (neutrophil count × monocyte count)/lymphocyte count.^22^^,^^23^

### Definition of covariates

This analysis incorporated comprehensive covariates encompassing sociodemographic traits, anthropometric measurements, lifestyle habits, and 3 common diseases. The covariates integrated into the analysis comprised age, gender, BMI, race, poverty income ratio (PIR), education levels, marital status, alcohol consumption, smoking status, hypertension, hyperlipidaemia, diabetes mellitus (DM), triglycerides (TG), high-density lipoproteins (HDL), and frequency of flossing or utilization of other cleaning devices. The study classified participants into 4 racial/ethnic categories: non-Hispanic white, non-Hispanic black, Hispanic, and other races. Using the Federal Poverty Level (FPL) with a threshold at 130%, we classified participants' socioeconomic status based on the PIR into 3 levels: low-income (PIR < 1.3), moderate-income (PIR 1.3-3.5), and high-income (PIR > 3.5). Educational attainment was stratified into 3 levels: less than high school, high school graduate, and more than high school. Three marital status groups were defined: married/living with partner, widowed/divorced/separated, and never married. Participants' smoking status was systematically defined based on a 100-cigarette lifetime consumption benchmark, distinguishing never, former, and active smokers.^24^ Alcohol consumption patterns were categorized as never, non-drinker, light drinker, moderate drinker, and heavy drinker.^25^ Participants were systematically categorized into weight status groups: underweight/healthy weight (BMI < 25 kg/m²), overweight (BMI 25-30 kg/m²), and obesity (BMI > 30 kg/m²).^26^ Detailed diagnostic criteria for hypertension, hyperlipidaemia, and diabetes mellitus were provided in Table S2. Oral hygiene practices were assessed by the frequency of dental floss use and cleaning device utilization, which were categorized into 3 levels: low (0-1 days/week), moderate (2-4 days/week), and high frequency (≥5 days/week).

### Statistical analyses

The analytical framework incorporated NHANES data, which employs a complex, hierarchical sampling design to maximize national demographic representativeness. The analysis incorporated data from 3 biennial survey cycles (2009-2014), with appropriate adjustment of weights. Data summarization utilized 2 distinct analytical approaches: categorical variables were quantified through frequency distributions and percentage representations, continuous variables through mean values and standard deviation calculations. Between-group comparisons (participants with versus without periodontitis) were conducted using Chi-square tests for categorical variables and ANOVA for continuous variables. Multicollinearity assessment was performed for BRI and all covariates using variance inflation factor (VIF) analysis, with values less than 3 indicating absence of multicollinearity (VIF > 10 suggests severe collinearity).^27^ All analyses were performed using R Studio (version 4.3.1) with the nhanesR package (version 0.9.4.3), following STROBE Guidelines for observational studies.

Indices (BRI, SII, and SIRI) underwent quartile-based stratification, systematically categorizing values from the lowest (Q1) to the highest (Q4) percentile ranges, as comprehensively illustrated in Table S3. The association between BRI and periodontitis was examined using weighted logistic regression through 3 models: an unadjusted model; Model 1 with adjustments for demographic factors (age, sex, and race/ethnicity); and Model 2 with comprehensive adjustments for demographics, BMI, socioeconomic factors (PIR, education, marital status), lifestyle behaviours (smoking, alcohol consumption, dental flossing), and clinical parameters (hyperlipidaemia, hypertension, DM, TG, HDL). The strength of associations was quantified using odds ratios (OR) with corresponding 95% confidence intervals (95% CI). To meet the assumptions of logistic regression, we assessed the linear relationship between continuous independent variables and the logit(p) transformation.

To investigate potential effect modification, subgroup analyses were performed across key covariates to examine the consistency of the BRI-periodontitis association within different subgroups. During initial data quality assessment, substantial missing data were identified for PIR (N = 850) and alcohol consumption (N = 534). To evaluate the robustness of our findings and address potential selection bias, we conducted sensitivity analyses on a larger sample (N = 9724) by excluding these variables with missing data. Additionally, we performed another sensitivity analysis using a dichotomized outcome classification (combining moderate/severe periodontitis versus no/mild periodontitis) through binary logistic regression to validate the primary findings.

The predictive performance of anthropometric indices (BRI, BMI, ABSI, and WC) for periodontitis was comprehensively examined utilizing Receiver Operating Characteristic (ROC) curve analysis. Between-model comparisons were conducted using Z-tests to assess differences in areas under the ROC curves (AUC).

The distribution pattern of BRI was visualized using histograms to assess normality (Figure 3A). To investigate potential nonlinear between BRI-periodontitis associations, Restricted Cubic Spline (RCS) analysis was performed with 3 strategically positioned knots, ensuring accurate capture underlying data trends.

By the 'Mediation' package, systematically evaluating the potential mediating mechanisms of SII and SIRI. Due to missing and outlier values in the blood cell count, the study population was reduced to 8381 participants**.** Figure S1 presents box plots showing the distribution of data for lymphocyte, monocyte, neutrophil, and platelet, as well as the exclusion of outliers in the blood cell count data. First, we examined the total effect of BRI on periodontitis without controlling for SII or SIRI (the c path in Figure 4A). Subsequently, linear regression models were fitted to evaluate the relationship between BRI and inflammatory mediators (SII or SIRI; a path). Subsequently, after controlling for SII or SIRI, multivariable logistic regression models were constructed to simultaneously estimate: (1) the residual direct effect of BRI on periodontitis (c' path), and (2) the effects of inflammatory indices on periodontitis (b path). The indirect effect was calculated by multiplying the a and b paths, and the mediation proportion was obtained by dividing the indirect effect by the total effect. Nonparametric bootstrapping with 500 iterations was employed to estimate the 95% confidence interval for the mediation proportion.

## Results

### Descriptive characteristics

The final analytical sample comprised 8415 participants (Figure 1). Baseline demographic and clinical characteristics were analysed according to periodontitis status (Table 1). Age-specific prevalence of periodontitis showed a significant increasing trend (*P* < .001), ranging from 28.9% in the 30-44 age group to 58.2% in those aged ≥65 years. Gender distribution analysis revealed that among the 8415 participants, 4129 (49.28%) were female and 4286 (50.72%) were male, with females showing significantly lower periodontitis prevalence than males (*P* < .001). BMI classification revealed that 26.34% (N = 2211) of participants were normal/underweight, 35.76% (N = 2910) were overweight, and 37.90% (N = 3294) were obese, with significant differences in periodontitis prevalence across weight categories (*P* = .010). Racial/ethnic disparities in periodontitis prevalence were observed (*P* < .001), with the highest rates among non-Hispanic black (56.94%), followed by Hispanics (54.18%), other races (45.19%), and lowest among non-Hispanic white (36.71%). Higher periodontitis prevalence was significantly associated with several sociodemographic and behavioural factors: marital status (widowed/divorced/separated), lower income (PIR < 1.3), lower educational attainment (less than high school), former alcohol consumption, current smoking status, and infrequent tooth brushing (0-1 days/week). Additionally, individuals with metabolic conditions (hyperlipidaemia, hypertension, and DM) showed higher prevalence of periodontitis. Anthropometric and metabolic parameters (BRI, WC, ABSI, BMI, TG, and HDL) differed significantly between periodontitis and non-periodontitis groups (all *P* < .01).

**Table 1:** Descriptive characteristics of the study population stratified by periodontitis.

Characteristic	Total	No periodontitis	Periodontitis	*P* values
	(N = 8415)	(N = 4178)	(N = 4237)	
BRI	5.44 ± 0.04	5.26 ± 0.05	5.69 ± 0.06	<.001
WC (cm)	100.30 ± 0.28	98.96 ± 0.31	102.20 ± 0.37	<.001
ABSI	0.08 ± 0.00	0.08 ± 0.00	0.08 ± 0.00	<.001
BMI (kg/m^2^)	29.18 ± 0.12	28.95 ± 0.14	29.51 ± 0.16	.005
TG (mmol/L)	1.81 ± 0.03	1.71 ± 0.03	1.95 ± 0.05	<.001
HDL (mmol/L)	1.38 ± 0.01	1.41 ± 0.01	1.34 ± 0.01	<.001
Age, n (%)				<.001
30-44	2978 (36.18)	1972 (71.10)	1051 (28.90)	
45-54	1893 (25.43)	953 (59.10)	940 (40.90)	
55-64	1750 (20.97)	687 (50.39)	1063 (49.61)	
≥65	1794 (17.42)	611 (41.80)	1183 (58.20)	
Sex, n (%)				<.001
Female	4129 (49.28)	2420 (66.28)	1709 (33.72)	
Male	4286 (50.72)	1758 (51.13)	2528 (48.87)	
Race/ethnicity, n (%)				<.001
Non‐Hispanic white	3878 (71.38)	2217 (63.29)	1661 (36.71)	
Non‐Hispanic black	1667 (9.74)	645 (43.06)	1022 (56.94)	
Hispanic	1952 (12.44)	819 (45.82)	1133 (54.18)	
Other race	918 (6.44)	497 (54.81)	421 (45.19)	
BMI, n (%)				.010
Underweight/Normal	2211 (26.34)	1157 (61.73)	1054 (38.27)	
Overweight	2910 (35.76)	1436 (58.77)	1474 (41.23)	
Obese	3294 (37.90)	1585 (56.26)	1709 (43.74)	
Marital status, n (%)				<.001
Married/living with partner	5491 (69.97)	2862 (61.88)	2629 (38.17)	
Never married	953 (9.92)	480 (55.00)	455 (45.00)	
Widowed/divorced/separated	1989 (20.11)	836 (48.95)	1153 (51.06)	
PIR, n (%)				<.001
<1.3	2426 (17.93)	898 (41.22)	1528 (58.78)	
1.3-3.5	3003 (34.21)	1363 (51.37)	1640 (48.63)	
>3.5	2986 (47.86)	1917 (70.28)	1069 (29.72)	
Education levels, n (%)				<.001
Below high school	705 (4.34)	189 (29.09)	516 (70.91)	
High school	2933 (30.49)	1106 (44.70)	1827 (55.30)	
Above high school	4777 (65.17)	2883 (67.06)	1894 (32.94)	
Alcohol consumption, n (%)				<.001
Never	1083 (9.74)	509 (56.47)	574 (43.53)	
Former	1437 (14.34)	569 (47.12)	868 (52.88)	
Mild	3061 (39.66)	1652 (62.83)	1409 (37.17)	
Moderate	1301 (18.08)	775 (66.00)	526 (34.00)	
Heavy	1533 (18.18)	673 (52.20)	860 (47.80)	
Smoking status, n (%)				<.001
Never	4630 (55.53)	2668 (67.20)	1962 (32.80)	
Former	2178 (26.95)	984 (54.66)	1194 (45.34)	
Now	1607 (17.52)	526 (37.39)	1081 (62.61)	
Hyperlipidaemia, n (%)				.004
No	2069 (24.55)	1113 (62.33)	956 (37.67)	
Yes	6346 (75.45)	3065 (57.38)	3281 (42.62)	
Hypertension, n (%)				<.001
No	4692 (59.60)	2638 (64.13)	2054 (35.87)	
Yes	3723 (40.40)	1540 (50.44)	2183 (49.56)	
Diabetes mellitus, n (%)				<.001
No	6115 (77.17)	3290 (62.10)	2825 (37.90)	
Prediabetes	720 (8.46)	327 (53.77)	393 (46.23)	
DM	1580 (14.37)	561 (42.62)	1019 (57.38)	
Flossing, n (%)				<.001
0-1 days a week	3211 (34.93)	1338 (51.04)	1873 (48.96)	
2-4 days a week	1989 (25.85)	1131 (65.16)	858 (34.84)	
≥5 days a week	3215 (39.22)	1709 (61.01)	1506 (38.99)	

BRI, body roundness index; WC, waist measurement; ABSI, A Body Shape Index; BMI, body mass index; TG, triglyceride; HDL, high density lipoprotein; PIR, poverty income ratio.

*P* value by chi-square test for classified variables.

### BRI and periodontitis risk correlation investigation

Binary logistic regression analyses demonstrated robust associations between BRI and periodontitis risk (Table 2). When analyzed as a continuous variable, BRI showed consistent significant associations across all models (unadjusted: OR = 1.09, 95% CI: 1.06-1.13, *P* < .001; adjusted model 1: OR = 1.08, 95% CI: 1.04-1.11, *P* < .001; fully adjusted model 2: OR = 1.08, 95% CI: 1.04-1.11, *P* = .030). Quartile analysis using Q1 as the reference, with adjusted Model 1 showing progressively higher odds ratios for Q2-Q4 (OR = 1.13, 95% CI: 0.97-1.33, *P* = .12; OR = 1.24, 95% CI: 1.04-1.48, *P* = .020; OR = 1.56, 95% CI: 1.56-1.86, *P* < .001, respectively). This pattern persisted in fully adjusted Model 2, with increasingly elevated risks across quartiles (Q2: OR = 1.33, 95% CI: 1.07-1.65, *P* = .010; Q3: OR = 1.48, 95% CI: 1.16-1.88, *P* = .004; Q4: OR = 1.70, 95% CI: 1.20-2.40, *P* = .010). Additionally, in all 3 models, the *P* for trend was statistically significant.

**Table 2:** Adjusted association of BRI with periodontitis.

Exposure	Unadjusted model	Adjust 1	Adjust 2
	Odds ratio (95% CI) associated with periodontitis		
BRI (continuous)	1.09 (1.06, 1.13); **<0.001**	1.08 (1.04, 1.11); **<0.001**	1.08 (1.04, 1.11); **0.030**
Quartile of BRI			
Q1	1 (Ref)	1 (Ref)	1 (Ref)
Q2	1.38 (1.18,1.61); **<0.001**	1.13 (0.97, 1.33); 0.120	1.33 (1.07, 1.65); **0.010**
Q3	1.63 (1.36,1.97); **<0.001**	1.24 (1.04, 1.48); **0.020**	1.48 (1.16, 1.88); **0.004**
Q4	1.82 (1.55,2.14); **<0.001**	1.56 (1.31, 1.86); **<0.001**	1.70 (1.20, 2.40); **0.010**
*P* for trend	<.001	<.001	.007

Unadjusted model: non-adjusted model.

Adjust 1: Adjust for age, sex, race.

Adjust 2: Adjust for age, sex, race, body mass index, poverty income ratio, education levels, marital status, smoking status, alcohol consumption, hyperlipidaemia, hypertension, diabetes mellitus, triglyceride, high density lipoprotein and flossing.

BRI, body roundness index; CI, confidence interval.

Bolded values indicate p-values <0.05.

### Receiver operating characteristic (ROC) curve

**Fig. 2:**
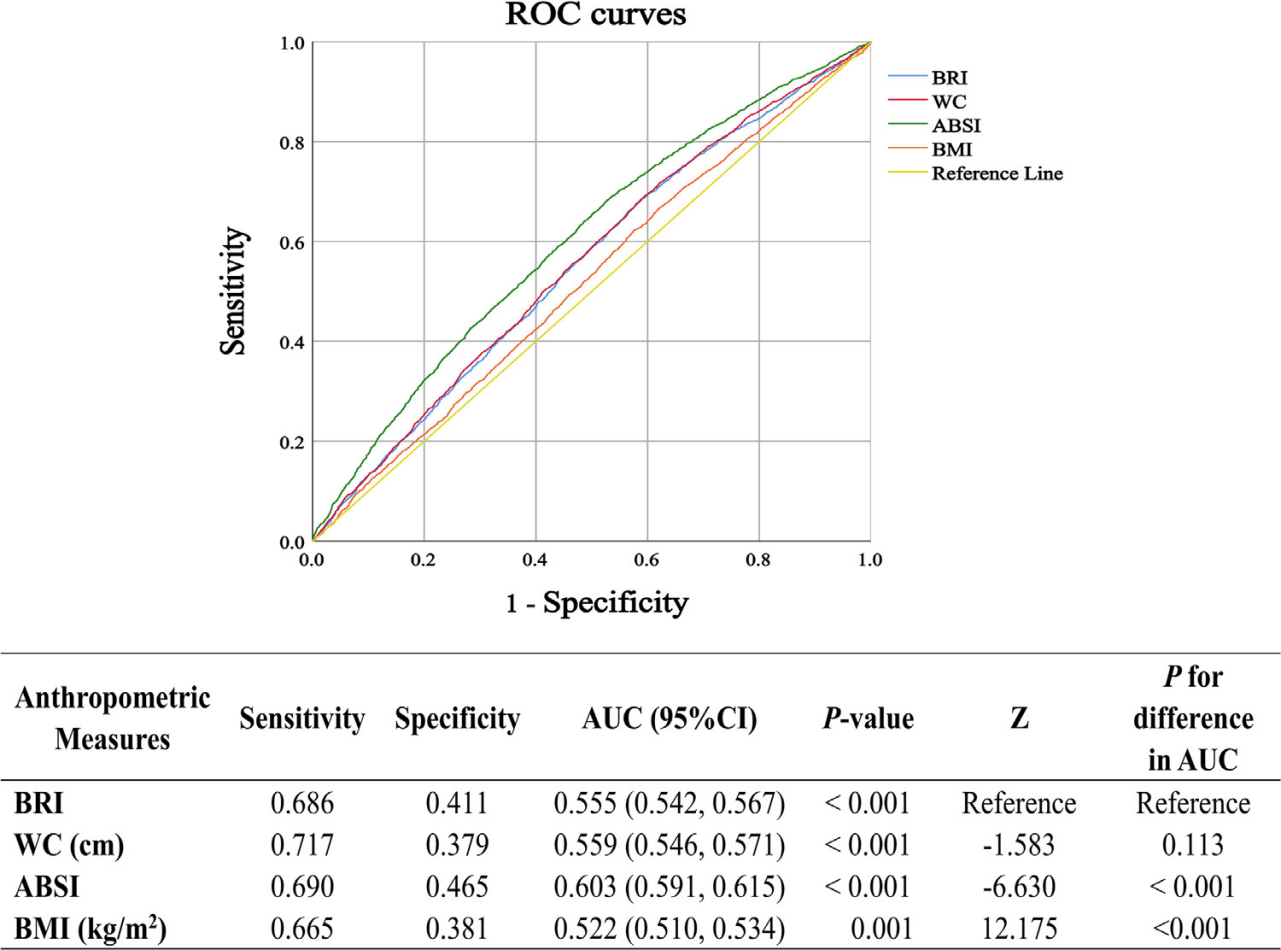
The receiver operating characteristic (ROC) curves for the prediction of periodontitis using body measurement indicators are presented. The Z-test of the area under the ROC curve (AUC) is used to assess the significant difference in predictive performance between the prediction models. BRI, body roundness index; WC, waist measurement; ABSI, A Body Shape Index; BMI, body mass index.

ROC curve analyses were performed to evaluate the predictive performance of various anthropometric indices (ABSI, BMI, WC, and BRI) in predicting periodontitis risk (Figure 2). All indices demonstrated significant diagnostic capability (AUC > 0.5) in unadjusted analyses. Pairwise comparisons revealed that BRI exhibited superior predictive performance compared to BMI (Z = 12.175, *P* < .001), comparable performance to WC (Z = -1.583, *P* = .113), but lower discriminative ability than ABSI (Z = -6.630, *P* < .001).

### Nonlinear relationships explore

**Fig. 3:**
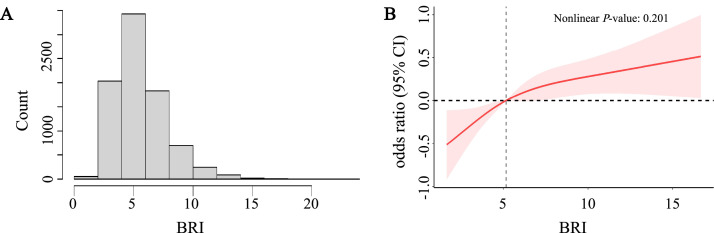
The distribution of BRI (A). The full-adjusted relationship between BRI and periodontitis using Restricted Cubic Spline (B). The solid line represents the fitted nonlinear curve. The area adjacent to the solid line represents the 95% confidence interval. BRI, body roundness index; CI, confidence interval.

In the fully adjusted model accounting for all potential confounders, the RCS analysis demonstrated no statistically significant nonlinear relationship (nonlinearity *P* = .201, Figure 3B). This finding corroborates the significant linear trend observed in the quartile analysis (Table 2).

### Subgroup analyses and sensitivity analysis

Subgroup analyses cross key demographic and clinical characteristics revealed differential associations between BRI and periodontitis risk. After comprehensive covariate adjustment, most subgroup analyses (sex, race, smoking status, hyperlipidemia, and DM) showed no significant effect modification (all *P* for interaction > .05; Table 3). However, significant effect modifications were observed for age and hypertension status (*P* for interaction = .017 and *P* for interaction < .001, respectively), with stronger associations noted among participants aged 30-44 years and those without hypertension.

**Table 3:** Adjusted association of BRI with periodontitis for subgroup analyses.

Subgroups	Q2	Q3	Q4	*P* for interaction
	Adjusted odds ratio (95% CI); P*			
Sex				.553
Female	1.19 (0.92, 1.53); .169	1.47 (1.03, 2.10); **.036**	1.83 (1.05, 3.21); **.036**	
Male	1.56 (1.08, 2.24); **.020**	1.53 (1.02, 2.30); **.043**	1.59 (0.95, 2.67); .076	
Age				.017
30-44	1.36 (0.95, 1.96); .091	1.91 (1.22, 2.99); **.007**	2.79 (1.50, 5.21); **.003**	
45-54	1.29 (0.84, 1.97); .235	1.47 (0.87, 2.49); .138	1.53 (0.67, 3.49); .293	
55-64	1.20 (0.61, 2.35); .580	0.97 (0.46, 2.05); .942	0.90 (0.42, 1.96); .787	
≥65	1.07 (0.68, 1.68); .776	1.19 (0.64, 2.22); .566	1.22 (0.56, 2.63); .603	
Race/Ethnicity				.587
Non‐Hispanic white	1.42 (1.08, 1.89); **.016**	1.47 (1.06, 2.04); **.023**	1.68 (1.03, 2.75); **.039**	
Non‐Hispanic black	1.29 (0.83, 1.98); .235	1.31 (0.78, 2.19); .279	1.65 (0.97, 2.80); .062	
Hispanic	1.08 (0.65, 1.80); .766	1.23 (0.60, 2.53); .552	1.42 (0.67, 3.03); .339	
Other race	1.01 (0.57, 1.77); .981	2.15 (1.03, 4.50); **.043**	2.05 (0.48, 8.75); .303	
Smoking status				.680
Never	1.36 (1.04, 1.79); **.029**	1.21 (0.82,1.79); .316	1.32 (0.77, 2.24); .291	
Former	1.29 (0.82, 2.01); .250	1.86 (1.25, 2.78); **.004**	2.40 (1.30, 4.45); **.008**	
Now	1.15 (0.64, 2.07); .620	1.45 (0.76, 2.77); .245	1.65 (0.79, 3.41); .168	
Hyperlipidaemia				.084
No	1.29 (0.83, 2.00); .236	1.37 (0.83, 2.28); .204	2.18 (1.11, 4.31); **.027**	
Yes	1.33 (1.06, 1.67); **.017**	1.45 (1.06, 1.98); **.023**	1.57 (1.04, 2.39); **.036**	
Hypertension				<.001
No	1.31 (1.00, 1.71); **.047**	1.31 (0.91, 1.89); .142	1.85 (1.10, 3.11); **.023**	
Yes	1.11 (0.72, 1.72); .607	1.50 (0.97, 2.33); .068	1.52 (0.89, 2.61); .121	
Diabetes mellitus				.185
No	1.30 (1.01, 1.66); **.040**	1.57 (1.20, 2.06); **.002**	2.00 (1.35, 2.96); **.002**	
Prediabetes	1.12 (0.47, 2.66); .781	0.84 (0.27, 2.63); .750	0.81 (0.20, 2.25); .758	
DM	1.97 (0.94, 4.12); .070	1.59 (0.72, 3.58); .243	1.40 (0.59, 3.32); .418	

⁎ We used the lowest quartile as the reference category. Adjust for age, sex, race, body mass index, poverty income ratio, education levels, marital status, smoking status, alcohol consumption, hyperlipidaemia, hypertension, diabetes mellitus, triglyceride, high density lipoprotein and flossing, but not for the specific stratification variables of interest. CI, confidence interval. Bolded values indicate p-values <0.05.

Sensitivity analyses using analytical approaches and alternative outcome definitions consistently supported the primary findings (Table S4 and Table S5). The results were generally consistent with those in Table 2, indicating that BRI (continuous) maintained significantly associated with the prevalence of periodontitis (unadjusted: OR = 1.10, 95% CI: 1.07-1.13, *P* < .001; adjusted model 1: OR = 1.08, 95% CI: 1.05-1.11, *P* < .001; fully adjusted model 2: OR = 1.09, 95% CI: 1.04-1.15, *P* = .002). The highest BRI quartile (Q4) showed particularly strong associations (fully adjusted model 2: OR = 2.00, 95% CI: 1.48-2.71, *P* < .001) compared to the lowest quartile (Q1). Similarly, when examining the role of BRI (continuous) in moderate/severe periodontitis, consistent associations persisted (unadjusted: OR = 1.09, 95% CI: 1.07-1.12, *P* < .001; adjusted model 1: OR = 1.07, 95% CI: 1.04-1.10, *P* < .001; fully adjusted model 2: OR = 1.07, 95% CI: 1.02-1.13, *P* = .010), with Q4 maintaining significantly elevated risk (fully adjusted OR = 1.92, 95% CI: 1.34-2.75, *P* = .001) compared to Q1. These comprehensive sensitivity analyses affirm the robustness of the observed associations between BRI and periodontitis risk.

### Systemic inflammation factors involved in BRI-periodontitis association

**Fig. 4:**
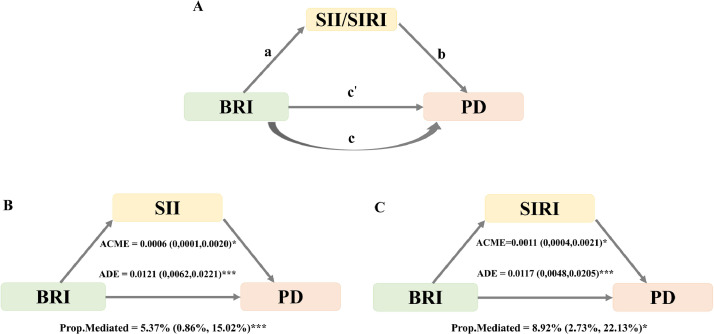
Mediation effects of SII and SIRI in the associations of BRI with periodontitis. Notes: Adjust for age, sex, race, body mass index, poverty income ratio, education levels, marital status, smoking status, alcohol consumption, hyperlipidemia, hypertension, diabetes mellitus, triglyceride, high density lipoprotein and flossing. BRI, body roundness index; PD, periodontitis; SII, systemic immune-inflammation index; SIRI, systemic inflammatory response index; ACME, average causal mediation effects (indirect effect); ADE, average direct effects. *P < .05, **P < .01, and ***P < .001.

In the mediation analysis, BRI, SII/SIRI, and periodontitis were treated as the independent variable, mediator variable, and dependent variable, respectively. As shown in Table 2, we have already demonstrated that the total effect of BRI on periodontitis (c path) is significant. The linear regression results in Table S6 show that the effect of BRI on SII or SIRI (a path) is significant in all models. The logistic regression results in Table S7 indicate that after including SII or SIRI, the effect of BRI on periodontitis (c’ path) and the effect of SII or SIRI on periodontitis (b path) are both significant in all models. Further investigation using mediation models, as shown in Figure 4, revealed significant mediation effects in the relationships between BRI and periodontitis risk through SII and SIRI. The mediation proportion for SII was 5.37%, and for SIRI was 8.92%.

## Discussion

This study found that higher BRI is significantly associated with an increased risk of periodontitis. Binary logistic regression analysis revealed that BRI was significantly associated with periodontitis across all adjusted models. Additionally, systemic inflammation, measured by the SII and SIRI, partially mediates the relationship between BRI and periodontitis. Compared to BMI, BRI demonstrated superior predictive performance for periodontitis, highlighting its potential as a more effective measure of obesity and oral health risk.

Our findings regarding sociodemographic and clinical risk factors align with and extend previous epidemiological evidence. Consistent with prior NHANES-based studies, we observed significant periodontitis risk gradients across various demographic and socioeconomic parameters, including age, gender, race/ethnicity, educational attainment, and economic status.^28^, ^29^, ^30^ The observed higher prevalence among older adults, males, non-Hispanic black, and individuals with lower socioeconomic status underscores the persistent influence of these fundamental determinants on periodontal health disparities. Furthermore, the observed associations with hyperlipidaemia, hypertension and DM align with previous systematic reviews and meta-analyses, suggesting common pathophysiological pathways.^31^, ^32^, ^33^ These findings emphasize the importance of considering these covariates when studying the relationship between BRI and periodontitis.

In recent years, the relationship between periodontitis and body measurements has garnered significant research attention. Body measurement indices, such as BMI, WC, and ABSI, including the BRI studied in this research, are characterized by their simplicity and accessibility, as they can be easily calculated by measuring basic body parameters. Moreover, these indices can reflect the extent of overall or abdominal obesity to some degree. Therefore, these body measurement indices are commonly used as risk factors for obesity-related diseases, such as DM, infertility, and cognitive impairment.^34^, ^35^, ^36^ Several studies have also explored the relationship between body measurement indices and periodontitis. Among these, BMI and WC are the most widely studied, with numerous studies showing a significant association between higher BMI and WC with an increased risk of periodontitis.^37^^,^^38^ Additionally, Gu et al found that in populations with hypertension, higher ABSI was significantly associated with an increased risk of periodontitis.^39^ As for BRI, past studies have explored its association with all-cause mortality, depression, and bone mineral density.^16^^,^^40^^,^^41^ However, to the best of our knowledge, this study is the first to investigate the relationship between BRI and periodontitis, and to examine the mediating effect of systemic inflammation in this relationship.

When explaining why a higher BRI increases the risk of periodontitis, we first need to discuss the origin and characteristics of the BRI. This index was initially proposed by Thomas et al in 2013 to develop a geometrical index of the human body using simple measures which are height and WC to reflect total and visceral body fat.^11^ Ultimately, the BRI was derived. Compared to BMI and WC, BRI improves the predictive accuracy for BF% and visceral adipose tissue. Both BF% and visceral body fat are strongly associated with periodontitis.^42^^,^^43^ This also helps explain the findings in our study, where BRI demonstrated better predictive performance for periodontitis compared to BMI, as shown by the ROC results. Obesity, particularly visceral obesity, has a significant impact on periodontitis through various mechanisms, including systemic inflammatory responses, alterations in the oral microbiome, elevated C-reactive protein levels, increased free fatty acids, insulin resistance, and oxidative stress.^13^ Among these mechanisms, systemic inflammatory responses play a central role. Obesity involves functional changes in adipose tissue, leading to the secretion of many pro-inflammatory factors. These pro-inflammatory factors circulate throughout the body, triggering systemic inflammatory responses,^12^ which have been proven to be a major risk factor for periodontitis.^19^ This pro-inflammatory property of adipose tissue may alter the immune microenvironment in periodontal tissues, disrupt local immune defence mechanisms, and thereby exacerbate inflammation and tissue destruction in periodontal regions.^13^ Visceral fat, in particular, exhibits higher metabolic activity, secreting not only a greater quantity of pro-inflammatory factors but also more potent ones.^44^ Based on this, BRI, as a sensitive indicator of visceral fat distribution, shows a significant association with the risk of periodontitis, even after controlling for various covariates, including BMI.

In this study, systemic inflammation was found to mediate the relationship between BRI and periodontitis. For the mediation effect to be valid, at least 3 conditions must be met: first, BRI must have a true effect on periodontitis prevalence, which has already been demonstrated in the previous section. Second, BRI must have an impact on systemic inflammation. Third, systemic inflammation must affect periodontitis. BRI provides a reliable reflection of BF% and visceral adipose tissue, and adipose tissue, particularly visceral fat, plays a crucial role in the systemic inflammatory response associated with obesity.^45^^,^^46^ Therefore, BRI may be associated with systemic inflammation by reflecting BF% and VAT. Additionally, our previous studies have shown that higher values of the SII or SIRI are significantly linked to an increased risk of periodontitis. The complex relationship between periodontitis and systemic inflammation has been widely studied and well-established in the literature.^7^^,^^47^

Our study's methodological robustness is primarily derived from its substantial sample size (N = 8415) and the utilization of nationally representative data from NHANES (2009-2014). The large-scale, population-based sampling strategy enhances the statistical power and generalizability of our findings. The application of survey weights in our analyses ensures that the results accurately reflect the demographic composition of the U.S. population, providing valuable epidemiological insights into the relationship between BRI and periodontal health at a national level.

However, several methodological limitations warrant consideration when interpreting these findings. The cross-sectional nature of the study design inherently precludes the establishment of causal inference between BRI and periodontitis. While our analyses demonstrate strong associations and potential mechanistic pathways, longitudinal studies are needed to confirm it. Additionally, the generalizability of our findings may be limited to the U.S. population, necessitating validation studies in other demographic and geographic contexts.

Currently, research mainly focuses on the direct association between BRI and periodontal disease, but it has not yet explored the multiple factors affecting BRI, which limits the understanding of the complex relationship between the two. Future studies could further investigate the influence of various factors such as genetics, nutrition, psychological factors, and lifestyle on BRI and its association with periodontal disease.^13^ From a genetic perspective, future research could explore specific genetic markers that are commonly associated with visceral fat distribution and periodontitis, which might represent shared risk factors for BRI and periodontal disease. Additionally, dietary habits and nutrients obtained from food play a significant role in both visceral obesity and periodontitis. Thus, examining how different dietary patterns regulate BRI-related inflammatory factors and their potential impacts on periodontal health could provide valuable insights. Furthermore, whether BRI is associated with psychological stress and whether stress management interventions could reduce BRI and the risk of periodontal disease warrants further exploration. Finally, lifestyle factors, such as sedentary behaviour, sleep habits, and neglectful attitudes toward oral health, might play a significant role in modulating BRI and periodontal health. These studies will help to more comprehensively understand the complex relationship between BRI and periodontal disease, providing scientific evidence for individualized prevention and treatment strategies.

## Conclusions

This study highlights a significant association between the BRI and periodontitis, with systemic inflammation playing a partial mediating role. BRI, as a simple and accessible measure, may be an effective tool for assessing periodontitis risk. The association between BRI and the risk of periodontitis is not merely a simple reflection of fat accumulation but is also closely related to complex inflammatory mechanisms triggered by adipose tissue dysfunction. These findings suggest that targeting BRI and systemic inflammation could help in preventing periodontitis. However, further studies are needed to confirm these results and explore causality.

## Data availability statement

The NHANES dataset is publicly available online, accessible at https://wwwn.cdc.gov/Nchs/Nhanes/Search/default.aspx.

## Ethics statement

This study was conducted under the auspices of the National Center for Health Statistics (NCHS), with comprehensive ethical oversight provided by the NCHS Institutional Review Board (IRB). Prior to data collection and health examinations, comprehensive informed consent was meticulously obtained from all eligible participants, ensuring full compliance with ethical research standards.

## Ethical approval

All the data used in our study were obtained from the National Health and Nutrition Examination Survey (NHANES). NHANES is a nationally representative cross-sectional study conducted under the direction of the National Center for Health Statistics (NCHS) to assess the health and nutrition status of the non-institutionalized population of the United States using a complex, multistage, and probabilistic sampling design. All of the surveys were authorized by the NCHS Ethics Review Board before being conducted, and all participants signed informed consent forms. More information is available at http://www.cdc.gov/nchs/nhanes/.

## Author contributions

ZH conceptualized the study design, conducted comprehensive data acquisition and interpretation, drafted the initial manuscript, and performed critical revisions. RZY contributed to the study's conceptualization, methodological design, and provided substantial critical manuscript revisions. PX contributed to the study's data acquisition and drafted the initial manuscript. GTL participated in study conception, refined the research design, and contributed to manuscript revision. All authors collectively reviewed and endorsed the final manuscript.

## Conflict of interest

None of the authors have any potential conflicts of interest.

## Appendix Supplementary materials

Image, application 1

Image, application 2

## Footnotes

Supplementary material associated with this article can be found in the online version at doi:10.1016/j.identj.2025.04.012.
