# Supplementary material for: Mediating Effects of Systemic Inflammation on the Association Between Body Roundness Index and Periodontitis in US Adults
Source: Int Dent J. 2025 Jun 10;75(4):100832. doi: 10.1016/j.identj.2025.04.012 (PMC12180997; doi:10.1016/j.identj.2025.04.012)
Supplement: Supplementary file 1 [file mmc1.docx]

All the data used in our study were obtained from the National Health and Nutrition Examination Survey (NHANES). NHANES is a nationally representative cross-sectional study conducted under the direction of the National Center for Health Statistics (NCHS) to assess the health and nutrition status of the non-institutionalized population of the United States using a complex, multistage, and probabilistic sampling design. All of the surveys were authorized by the NCHS Ethics Review Board before being conducted, and all participants signed informed consent forms. More information is available at <http://www.cdc.gov/nchs/nhanes/>.
